# Correlation between Fracture Morphology and Microstructural Evolution during Long-Term Aging of EK61 Superalloy

**DOI:** 10.1155/2020/1087024

**Published:** 2020-04-23

**Authors:** Jin Huang, Guohua Xu, Heyong Qin, Lei Zheng

**Affiliations:** ^1^School of Materials Science and Engineering, University of Science and Technology Beijing, Beijing 100083, China; ^2^High-Temperature Materials Department, China Iron & Steel Research Institute Group, Beijing 100081, China

## Abstract

Microstructural evolutions of EK61 superalloy during long-term aging until 1000 h at 700°C and 750°C, respectively, are studied by combination of Scanning Electron Microscope (SEM) and Transmission Electron Microscope (TEM). Impact fracture morphologies after aging for different time are observed by the SEM. The microstructure is found to be relatively stable during aging at 700°C, and the fracture morphologies are characterized by transgranular fracture. At 750°C, the coarsening of *γ*′ phase leads the reduction of the quantity of dimples, the chainization of carbides on grain boundaries leads to intergranular fracture, and the netting of *η* phases within grains leads to the formation of lamellar cleavage steps. It is obvious that the destabilization of precipitated phases affects fracture morphology significantly. The relationship between fracture morphology and the microstructure promotes the evaluation of service reliability of EK61 superalloy.

## 1. Introduction

Nickel-based superalloy EK61 can maintain good stability and excellent mechanical properties in the range of -253°C to 750°C. At present, EK61 superalloy is mainly used in rocket engine turbine disk [1–6]. As well known, the service conditions of the engine turbine disk are quite harsh. The disk often works in the oxygen-enriched gas environment of high pressure and large flow rate and also needs to bear high stress cycle load. In order to ensure the reliability of the engine turbine disk for a long time in such an environment, the EK61 alloy must have excellent comprehensive mechanical properties. Impact toughness is one of the important properties [7], which can be used to evaluate the toughness and brittleness of the alloy and to reveal the brittle fracture tendency of the material [8].

Impact fracture morphology can show the fracture mechanism intuitively, which is very helpful to the failure analysis [9]. It is obvious that fracture morphology is related directly to microstructure [10]. From the point of the growth of *η* phase and the coarsening of *γ*′ phase during aging, Zhao and Xie [11] analyze the formation process of brittle impact fracture of a new nickel-based superalloy. Claudio Gennaria et al. [12] found that the precipitation of a small quantity of phases with different morphologies in UNS S32205 steel decreased the impact properties of the alloy.

On the basis of previous studies, the nickel-based superalloy EK61 is aged for a long time at different temperatures. The microstructure and impact fracture morphology during aging are observed and analyzed. The effect of microstructure degradation on ductile-brittle transition of EK61 superalloy during long-term aging is studied, which provides a basis for failure analysis and safety evaluation.

## 2. Materials and Methods

The experimental material used in this paper is the forged EK61 alloy. The chemical composition is shown in Table 1.

The alloy is first heat-treated at 980°C × 1 h/water quench + 730°C × 15 h/water quench + 650°C × 10 h/water quench. Then the aging is carried out at 700°C and 750°C for 30 h, 100 h, 200 h, 500 h, and 1000 h, respectively. After aging, the samples are polished and corroded. The erosion regime is 20%H_2_SO_4_ + 80%CH_3_OH, 20-25 V voltage, 20-25 s erosion time, and 150 ml H_3_PO_4_ + 10 ml H_2_SO_4_ + 15 gCrO_3_, voltage 3-4 V, 5-7 s erosion time.

The precipitated phases are observed by Field-Emission Scanning Electron Microscope (FE-SEM) and identified by Scanning Electron Microscope-Energy Dispersive Spectrometer (SEM-EDS) and TEM. The impact test is carried out at room temperature, using a JB-30B Charpy impact machine with impact energy of 0~300 J, and the pendulum falling speed is 5.2m·s^−1^. The dimension of the specimens is 55 mm × 10 mm × 10 mm. There is a V-groove in the middle of the specimen with 2 mm depth. The fracture surface is protected and then cut by wire cutting machine. The fracture samples are cleaned ultrasonically for 15 minutes and then observed by the SEM after drying.

## 3. Results and Discussion

### 3.1. Evolution of Impact Property after Long-Term Aging at Different Temperature

Table 2 lists the impact property at room temperature of EK61 superalloy after long-term aging at 700°C and 750°C. It can be seen that the impact property gradually decreases with increased aging time.

### 3.2. Evolution of Microstructure during Long-Term Aging

**Figure 1:**
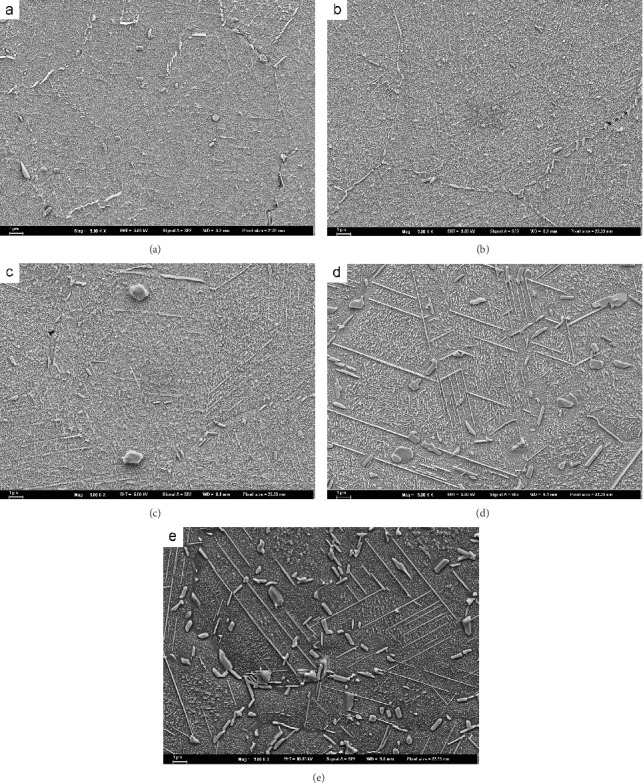
Microstructure after aging at 700°C for different time: (a) 30 h, (b) 100 h, (c) 200 h, (d) 500 h, and (e) 1000 h.

**Figure 2:**
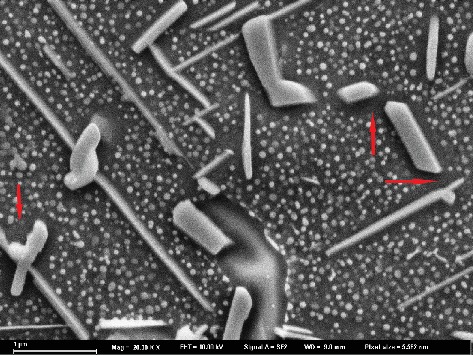
*γ*′ phase depletion zones around needle-like phases aging for 1000 h at 700°C.

Figure 1 shows the microstructure evolution of EK61 superalloy during aging at 700°C. The average sizes of *γ*′ phases in bulk after aging for different time are obtained and listed in Table 3, which displays that *γ*′ phase grows gradually during aging for 30-200 h. At the same time, it can be seen that the morphologies of *γ*′ phases and carbides at grain boundaries keep almost unchanged. In addition, a needle-like phase starts to appear after aging for 30 h and becomes more obvious as time extends. During aging from 500 h to 1000 h, the average sizes of *γ*′ phases in bulk increase still gradually. The size of carbides at grain boundaries becomes larger, and the shape changes from short slice to short bar. The needle-like phase becomes longer and thicker. It is noteworthy that there are obvious *γ*′ phase-depleted zones around the needle-like phases and carbides, as indicated by the arrows in Figure 2, which proves the precipitating of needle-like phases to be at the expense of *γ*′ phases. During long-term aging, thermodynamically, the *γ*′ phases became metastable, and therefore transform to the more stable needle-like phases [13]. From Figure 1, it can be concluded that the main precipitates are *γ*′ phases, needle-like phases, and carbides. During aging, the microstructure degenerates gradually.

**Figure 3:**
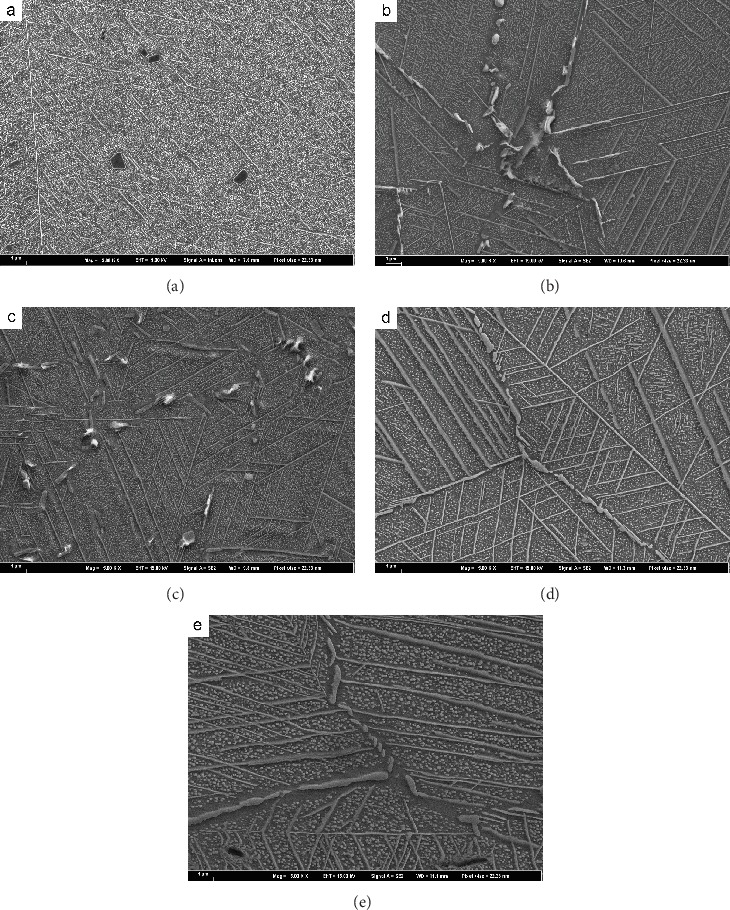
Microstructure after aging at 750°C for different time: (a) 30 h, (b) 100 h, (c) 200 h, (d) 500 h, and (e) 1000 h.

**Figure 4:**
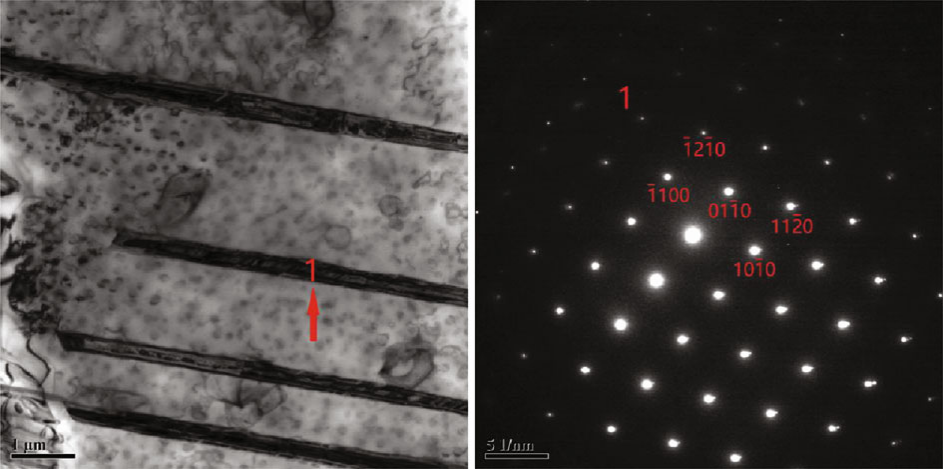
TEM morphology and calibration of SADPs of *η* phase aging for 1000 h at 750°C. $\left[2\bar{1}\bar{1}0\right]\eta //\left[1\bar{1}0\right]\gamma$.

Figure 3 shows the microstructure evolution of EK61 superalloy during aging at 750°C. The average sizes of *γ*′ phases in bulk after aging for different time are also obtained and listed in Table 3, which displays that *γ*′ phase grows continuously during aging until 1000 h, and the average size is larger than that at 700°C for a certain aging time. During aging, the carbides at grain boundaries change from discontinuous short rod and block to continuous chains. The long needle-like phases are small, scattered, and short at the beginning. They evolve form the larger dendritic shape gradually and form a net after 500 h aging that covers the entire grain. EDS measurements listed in Table 4 show that the long needle-like phases are Ni_6_(Al, Ti, Nb) after 500 h aging and determined to be *η* phases by TEM lattice calibration (as indicated by the arrows in Figure 4). The orientation relation between *η* phase and bulk is also shown in Figure 4. It can be concluded from Figure 3 that the main precipitates are *γ*′ phases, *η* phases, and carbides either. Besides, the microstructure degenerates during aging as well. The higher the aging temperature is, the more serious the degradation is.

### 3.3. Impact Fracture Morphology

**Figure 5:**
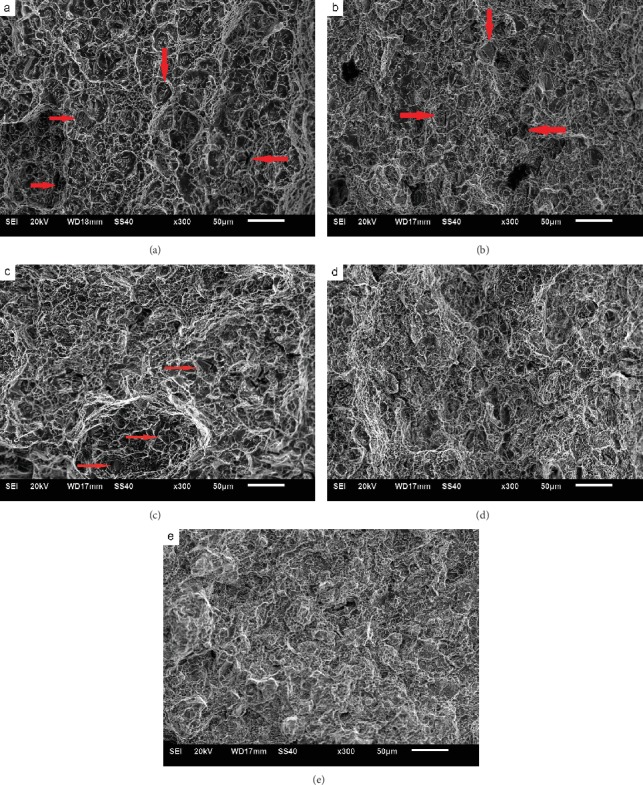
Impact fracture after aging at 700°C for different time: (a) 30 h, (b) 100 h, (c) 200 h, (d) 500 h, and (e) 1000 h.

Figure 5 shows the impact fracture morphology after aging at 700°C for different time. When aged for 30-200 h, the fracture surface morphology shows dimples mainly and broken carbides can be seen at the bottom of several dimples (as indicated by the arrows). With the extension of aging time to 500 h, dimples become shallow and small. Moreover, tearing edges appear and the quantity of dimples on the fracture surface decreases. When aging for 1000 h, dimples become even shallower and smaller. At the same time, the amount of tearing edges increases. It is clear that the main feature of fracture morphology is dimple and tearing edge even after aging of 1000 h, and the fracture is still transgranular ductile mode.

**Figure 6:**
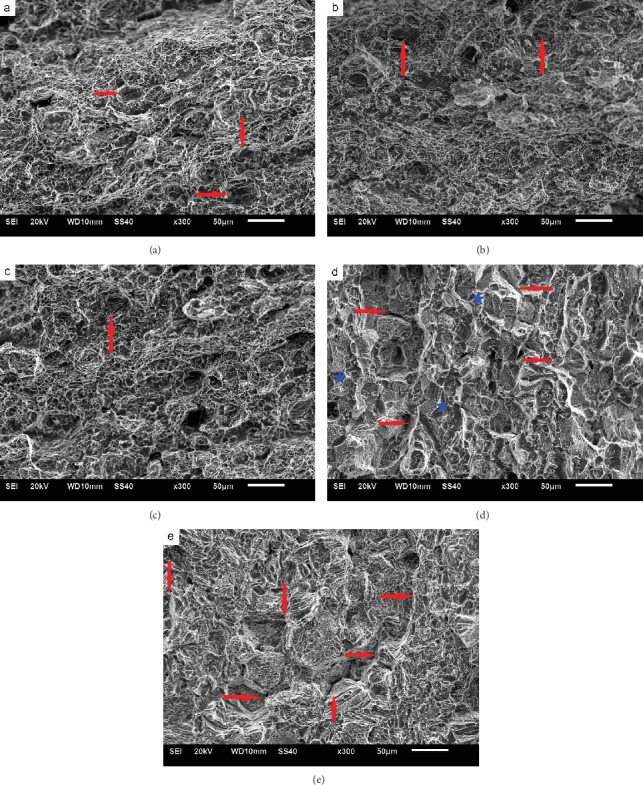
Impact fracture after aging at 750°C for different time: (a) 30 h, (b) 100 h, (c) 200 h, (d) 500 h, and (e) 1000 h.

Figure 6 shows the impact fracture morphology after aging at 750°C for different time. During aging for 30-200 h, the fracture morphology shows dimples mainly, and the fracture mode is transgranular. The dimples are small and shallow. Broken carbides can also be seen at the bottom of several dimples (as indicated by the arrows). After aging for 500 h, the fracture morphology changes significantly. The dimples on the fracture surface are seldom observed while short and bending tearing appears obviously. Furthermore, the fracture surface begins to show the characteristics of intergranular fracture (as indicated by the arrows), and lamellar cleavage steps appear within the grains (as indicated by the stars). After aging for 1000 h, the fracture surface exhibits evident intergranular fracture characteristics (as indicated by the arrows) and is full of lamellar cleavage steps within the grains, which demonstrates the fracture to be significantly brittle.

## 4. Discussion and Analysis

It can be seen that both the aged microstructure and the fracture morphology take significant changes at 750°C while they are relatively stable at 700°C, indicating that the change of aged microstructure influences the fracture morphology directly [14–16].

It is well known that *γ*′ phase is harder than the matrix. In the process of deformation, *γ*′ phase is difficult to deform, which leads to the formation of a weak zone interface between *γ*′ phase and matrix. In this case, microcracks are easily initiated at the *γ*/*γ*′ interface [17]. At the crack tip, the stress state is basically triaxial. Thus, the microcrack will form a small plastic pit with *γ*′ phase in the grain as the core. Afterwards, it forms dimples representing the morphology of the ductile fracture. Therefore, the density, depth, and distribution of dimples depend on the number, size, and distribution of *γ*′ phases, respectively [15]. During the long-term aging at 700°C and 750°C, *γ*′ phases only coarsen and the morphology keeps granular. Since the size of *γ*′ phase increases and the volume fraction decreases, the size and the quantity of dimples on the fracture surface decrease.

Carbides within the bulk will also induce dimples when the local stress is over the strength of carbides. Similarly, a microcrack initiates and thus a small plastic pit will develop into a dimple after fracture. However, because the amount of carbides within the bulk is much less than that of *γ*′ phases, the density, depth, and distribution of the dimples are seldom affected by carbides within the bulk. As for the carbides at grain boundaries, they play two roles affecting mechanical properties of superalloy according to different morphologies [18]. When the carbides are dispersed in grain boundaries, they can not only strengthen the grain boundary by increasing the difficulty of grain boundary slipping but also pin the grain boundaries to inhibit the grain coarsening under high temperature. In this case, the effect of carbides is positive [19]. Whereas when carbides are distributed at grain boundaries in the form of continuous flakes, dislocations are blocked at the interface between carbides and matrix *γ*, resulting in stress concentration. In addition, the coarsening of grain boundary carbides will consume strengthening elements (Ti, Mo, Nb, etc.) and leads to the formation of weak areas at near grain boundaries. Under this circumstance, the stress concentration occurs easily at the carbides when the alloy is impacted and thus causes microcrack initiation. Subsequently, microcracks connect continuously and bring out intergranular fracture finally. As a result, the chainization of carbides at grain boundaries leads to intergranular fracture and grain boundary embrittlement during impact. During the aging process at 700°C (see Figure 2), the carbides do not form a chain and thus the impact fracture mode is always transgranular. From Figure 3, it is seen that aging at 750°C for 30 h, the carbides precipitate discontinuously at grain boundaries. After 500 h aging, carbides form a chain nearly and the fracture surface begins to show intergranular fracture characteristics. After aging for 1000 h, the carbides at grain boundaries form obvious chains, and the impact fracture morphology is mainly intergranular fracture.

It is obvious that *η*-Ni_6_(Al, Nb, Ti) phases [20] grow in a way of needle expansion and present the distribution of array arrangement during aging. The growth of *η* phase consumes elements of Al, Ti, and Nb that form *γ*′ phases and thus results in *γ*′ phase depletion zone around the *η* phase. The strength of this depletion zone is thus low due to the lack of *γ*′ hardening. During deformation, dislocations are obstructed by *η* phases and accumulated in front of *η* phases. Since the depletion zone is relatively weak, the microcrack will emerge. From Figure 3, it is known that short rod *η* phases precipitate visibly within grains after aging at 750°C for 30 h, and the distribution of array arrangement can be observed. However, the fracture morphology shows obvious lamellar cleavage only after aging for 500 h (see Figure 6). It is notable that the thick *η* phases form a network structure after aging for 500 h and covering the whole grains with the aging time up to 1000 h. Therefore,it is the netting of *η* phases that leads to the formation of intergranular lamellar cleavage steps. After aging at 700°C for 1000 h, the degree of growth of *η* phase is similar to that aged at 750°C for 200 h. There is no cleavage step on the fracture surface during aging at 700°C, which further indicates that the netting of *η* phases induces the cleavage fracture morphology.

## 5. Conclusions

Microstructure degenerated clearly during aging at 750°C, which is indicated by the coarsening of *γ*′ phases, the chainization of carbides at grain boundaries, and the netting of *η* phases. while the microstructure during aging at 700°C is relatively stable, which is indicated by the less size of *γ*′ phases, the less amount of *η* phases, and the unchained distribution of carbides at grain boundaries.

Microstructure degradation is the main reason for the change of fracture morphology. The coarsening of *γ*′ phases leads to the decrease in the quantity of dimples, the chainization of carbides at grain boundaries leads to the intergranular fracture, and the netting of *η* phases leads to the formation of lamellar cleavage steps.

## Figures and Tables

**Table 1:** Chemical composition of EK61 superalloy, wt. %.

Element	C	Cu	Al	Fe	Cr	Ti	Mo	Nb	Si	Mn	S	P

Content	0.05	0.5	1	14	16	0.5	4	4.5	≤0.2	≤0.1	≤0.004	≤0.009

**Table 2:** Impact property at room temperature of EK61 superalloy after long-term aging.

Aging time (h)	Aged at 700°C		Aged at 750°C	
	Akv (J)	Error	Akv (J)	Error
30	49.0	±7.08	47.0	±7.37
100	37.0	±3.06	37.5	±9.09
200	38.5	±2.75	32.0	±10.04
500	27.5	±1.89	30.0	±8.01
1000	16.5	±3.21	26.0	±2.25

**Table 3:** Diameter of *γ*′ phases after long-term aging.

Temperature (°C)	700					750				
Time (h)	30	100	200	500	1000	30	100	200	500	1000
Diameter (nm)	21.66	25.93	31.93	36.86	44.12	27.02	38.84	41.95	55.24	72.60
Error (nm)	0.129	0.301	0.082	0.186	0.132	0.047	0.091	0.143	0.218	0.094

**Table 4:** EDS results of needle-like phases after aging 1000 h at 750°C.

Element	Al	Ti	Cr	Fe	Ni	Nb	Mo
Wt. %	1.70	1.04	11.42	8.66	60.75	12.64	3.80
At. %	3.78	1.30	13.15	9.29	61.97	8.15	2.37

## Data Availability

All the data used to support the findings of this study are included within the article.
